# Pollutants’ Release, Redistribution and Remediation of Black Smelly River Sediment Based on Re-Suspension and Deep Aeration of Sediment

**DOI:** 10.3390/ijerph14040374

**Published:** 2017-04-01

**Authors:** Lin Zhu, Xun Li, Chen Zhang, Zengqiang Duan

**Affiliations:** State Key Laboratory of Soil and Sustainable Agriculture Institute of Soil Science, Chinese Academy of Sciences 71 East Beijing Road, Nanjing 210008, China; xli@issas.ac.cn (X.L.); zhangcissas@163.com (C.Z.)

**Keywords:** re-suspension, contaminated sediment, pollutants distribution, in situ remediation

## Abstract

Heavily polluted sediment is becoming an important part of water pollution, and this situation is particularly acute in developing countries. Sediment has gradually changed from being the pollution adsorbent to the release source and has influenced the water environment and public health. In this study, we evaluated the pollutant distribution in sediment in a heavily polluted river and agitated the sediment in a heavily polluted river to re-suspend it and re-release pollutants. We found that the levels of chemical oxygen demand (COD), NH_4_^+^-N, total nitrogen (TN), and total phosphorus (TP) in overlying water were significantly increased 60 min after agitation. The distribution of the pollutants in the sediment present high concentrations of pollutants congregated on top of the sediment after re-settling, and their distribution decreased with depth. Before agitation, the pollutants were randomly distributed throughout the sediment. Secondly, deep sediment aeration equipment (a micro-porous air diffuser) was installed during the process of sedimentation to study the remediation of the sediment by continuous aeration. The results revealed that deep sediment aeration after re-suspension significantly promoted the degradation of the pollutants both in overlying water and sediment, which also reduced the thickness of the sediment from 0.9 m to 0.6 m. Therefore, sediment aeration after suspension was efficient, and is a promising method for sediment remediation applications.

## 1. Introduction

Heavily polluted sediments in rivers have gradually become a significant factor in water pollution, especially in developing countries [1]. Since the water in urban rivers is relatively static, the concentrations of pollutants in the sediment, such as organic matter, ammonium nitrogen, phosphorus, and heavy metals are significantly higher than in the overlying water [2,3,4,5,6,7]. Sediment and overlying water are continuously exchanging material and energy, so that the pollutants maintain a dynamic equilibrium of adsorption and release [8]. If environmental conditions are changed by external factors, such as storms or human activities [9], the pollutants are released into the overlying water through desorption, dissolution, and biological decomposition, causing secondary pollution, and the sediment changes from being a pollution adsorbent to a release source and influences the water environment and public health [10].

Another important aspect of river sediment pollution is that the deposition distribution is disordered from high pollutant loading, different rates of adsorption, and from the continuous input of the pollutants [11,12]. Normal river sediment is formed by a long process of natural subsidence and accumulation, and can be generally divided into four layers, from the top to bottom: floating mud, flowing mud, silt, and silt soil. The adsorption of contaminants in sediment follows the Langmuir Adsorption Isotherm [13]. Floating mud and flowing mud are the major pollutant adsorbing layers in river sediment, in the first 0 cm to 80 cm of the sediment [14]. This section of the sediment has a moisture content of about 85%–95%, and the particle size is small (0.01–0.05 mm). Because this section has the highest pollutant adsorption capacity and adsorption rate of all river sediment sections (adsorption may reach saturation in 4 to 8 h), and the lowest density (wet density) is 1.05–1.7 g/cm^3^, it can easily be disturbed. On the contrary, silt and silt soil are large particles, such as grains of sand, with a lower moisture content (<60%), a higher density (wet density is 1.05–1.7 g/cm^3^), and a significantly lower pollutant adsorption capacity and adsorption rate, as opposed to the floating mud and flowing mud [15,16]. In this respect, silt and silt soil are difficult to disturb. Similar studies found that river sediment with high oxygen consumption often contained a greater portion of colloidal and flocculent components [17]. Utley et al. proposed that oxygen consumption in the sediment is inversely related to the sand content in the sediment, after researching the sediment oxygen demand (SOD) of the Arrapkha River [18]. These studies suggested that the higher oxygen consumption in the sediment resulted from the colloidal and floc particles at the bottom of the soil, which easily adsorb organic particles in the water. Such a distribution of pollutants is also commonly observed in healthy river channels, as well as in the sediment of large lakes. In heavily polluted rivers, the continuous and high intensities of pollution load within short time frames cause pollution that cannot deposit in the normal distribution of the sediment, and form a random distribution of polluted sediment, especially when there is not enough time for the large particles to settle and be doped in the colloidal and floc sections. As such, the pollution is distributed in the density gradient, with unusual pollution concentration and release processes. Therefore, the re-suspension of contaminated sediments has received extensive attention [19], especially regarding the release of heavy metals [20], phosphorus [15], and organic matter [21], as well as the contaminant bioavailability and toxicity. The re-suspension method in situ could significantly reduce the concentration of contaminants without removing highly contaminated sediments. Additionally, the costs for dewatering, transportation, and tipping when handling the contaminated disposal materials can be reduced. Traditional sediment remediation techniques (including shallow sediment or overlying water aeration) [11,22] and natural events (waves and storms) only affect the surface sediment, but pollutants will continuously be released from the deep sediment to the surface and overlying water.

In this study, sediments were re-suspended by agitation, pollutants were released in the re-suspension process, and the distribution of the sediment pollutants after settling was investigated. Then, deep sediment aeration equipment was used to re-suspend the sediment. The agitation significantly promoted the release of pollutants in the sediment, and changed the distribution of the pollutants in the sediment. The changes of the reactant concentrations through the re-suspension process dramatically improved the efficiency of the degradation of the pollutants. This study provides a valuable method for the remediation of heavily polluted river sediments.

## 2. Materials and Methods

### 2.1. Site Description

The heavily polluted river channel (Feng-gang River) was located in Foshan City in the Guangdong province (from 112°58’31″E to 113°06′02″E and from 23°01′52″N to 23°04′2″N). The basic characteristics of this climate are warm and rainy, and the annual average temperature is between 21.2–22.2 °C. The river is 1.2 km long, 25 m in average width and 1.2 m in depth. The sediment thickness averaged 1 m. The river is mostly surrounded by farmland, residences, small farms, and a few leather-processing factories. There are about 12 outfalls along the coasts, and the main pollutants are nitrogen, phosphorus, and organic matter. The river has heavy organic pollution and severe eutrophication, consistent with observations that the water is black, with no aquatic animals or plants.

### 2.2. Sediment Disturbance and Deep Aeration

A 10-m by 4-m rectangular region was sectioned off by a rubber wall in the experimental river channel.

Disturbance: Hard plastic blast pipes of coarse diameters (10 cm) connected with air blowers were inserted into the deep sediment core (1.2 m). Vigorous air was blown through the hard plastic pipes for 1 h to achieve sediment suspension.

Deep aeration: The sediment aeration equipment (a micro-porous air diffuser) was installed during the sedimentation process (after stopping the disturbance for 10 min). Intermittent aeration was maintained for 15 days, for 1 h every 2 h.

### 2.3. Water, Sediment Sampling, and Analysis

Water samples were collected in 350 mL polypropylenes bottles mid-stream at 0.2 m below the water surface, and were analyzed in triplicate. The bottles were completely filled with water without bubbles and headspace, were immediately placed in an icebox at 4 °C, and the samples were analyzed within 24 h. The chemical oxygen demand (COD), ammonium nitrogen (NH_4_^+^-N), nitrate nitrogen (NO_3_^−^-N), total nitrogen (TN), phosphate (PO_4_^−^-P), and total phosphorus (TP) of each water sample were measured with the methods specified in the standard methods for the examination of water and wastewater [23]. Dissolved oxygen (DO) was measured using a handheld dissolved oxygen instrument (PD320, Alalis, Shanghai, China). The purity levels of all chemical reagents used in the analysis were analytical reagents or better, and the analytical precision was within 5%. 

The sediment was collected without disturbance using a columnar sampler made by a steel pipe divided into two sections, with a hinge pin to ensure that the sampler could be opened and closed. The sampler was vertically inserted into the mud, and a hammer was used to push the pin into the deep clay. Sediment samples were first filtered to remove stones, shells, weeds, and other impurities, and were then dried in the air and sieved with a 20-mesh sieve repeatedly until all soils passed through the sieve.

The particle size of the suspended sediment samples, which was determined using a Microtrac S3500 Laser Particle Size Analyzer (Microtrac Inc., Montgomeryville, PA, USA) based on the suitable standard operation procedure (refractive index, 1.59; measurement time, 20s; flow, 90%), was classified into four categories: 2 μm, 2–50 μm, 50–100 μm, and 100–2000 μm [24].

The total organic carbon (TOC) and total nitrogen (TN) were determined using a total organic carbon and total nitrogen analyzer (Multi C/N 3000, Analytik Jena AG, Jena, Germany). The TOC of the sediment samples were washed with concentrated sulfuric acid and potassium dichromate. The total nitrogen (TN) of the samples underwent acidosis by hydrochloric acid and hydrofluoric acid, and then digestion in concentrated sulfuric acid. The TP of the samples underwent digestion in sulfuric acid and perchloric acid. The total phosphorus (TP) of the sediment was tested by semi-micro Macro Kjeldahl and by antimony molybdenum spectrophotometry.

## 3. Results and Discussion

### 3.1. The Distribution of Pollutants in the Sediment

**Figure 1:**
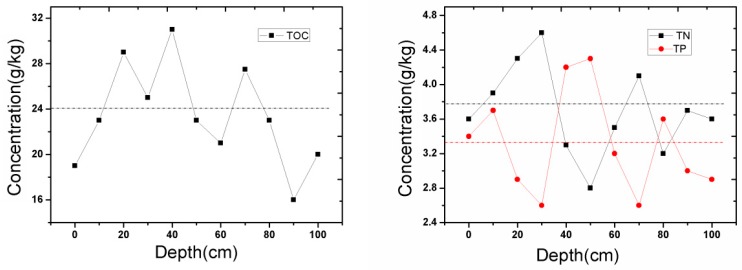
The total organic carbon (TOC), total nitrogen (TN), and total phosphorus (TP) distribution of the sediment at depths of 0–100 cm (the dotted line represents the average value).

The distribution of TOC, TN, and TP are shown in Figure 1. The TOC levels increased at depths of 0–20 cm, were slightly reduced at 30 cm, increased to a maximum value of 30.6 g/kg at depths of 40–50 cm, and dramatically reduced to 20.2 g/kg at a depth of 60 cm. Until the depth increased to 100 cm, the distribution of the TOC showed a random distribution with no obvious regularities. TN and TP also showed no obvious regularities of distribution with the depth.

### 3.2. Disturbance of Water Contaminants after Severe Disturbance

**Figure 2:**
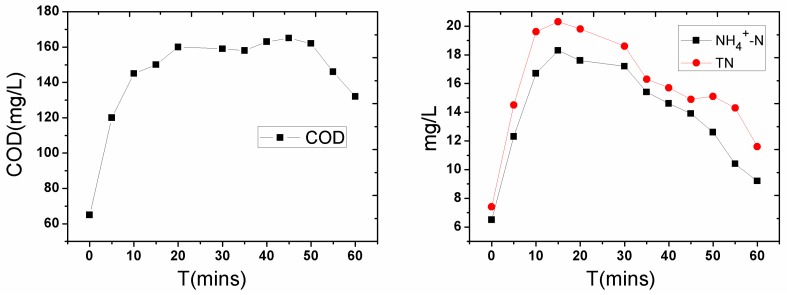
The chemical oxygen demand (COD), ammonium nitrogen (NH_4_^+^-N), and total nitrogen (TN) contents in overlying water during the re-suspension process of the sediment.

After agitation, the COD in the overlying water increased dramatically from 65 mg/L to 160 mg/L at 20 min, plateaued between 20–45 min, and slightly reduced after 45 min (Figure 2). The release of organic matter into the overlying water at the beginning of the disturbance caused a dramatic rise of the COD. When the aeration disturbance process completed, the release of organic matter decreased, and a portion of the organic matter was oxidatively degraded and caused the decline of the COD after about 40 min. The disturbance effect of the aeration influenced the peak release of the organic matter within 40 min. This is because part of the volatile organic compounds volatilized out of the water during the disturbance process, and a portion of the organic matter was oxidatively degraded [25].

NH_4_^+^-N and TN showed similar trends compared to those of the COD, except that the maximum value was reached earlier (between 15–20 min), because NH_4_^+^-N had the characteristics of slow adsorption and quick release in the sediment. The NH_4_^+^-N increased from 6.3 mg/L to 18 mg/L, and TN increased from 7.2 mg/L to 20.3 mg/L, with a shorter platform in this time period (15–30 min), and then significantly reduced after 45 min, as shown in Figure 2. This is mainly because part of the ammonia nitrogen was directly blown off in the form of ammonia during the intense aeration process [26,27].

**Figure 3:**
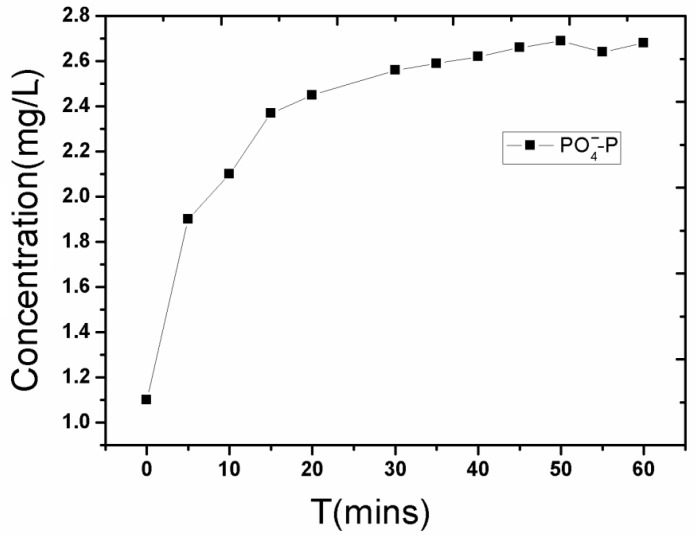
Changes of phosphate (PO_4_^−^-P) contents in the overlying water during the re-suspension process of the sediment.

The content of soluble phosphate continuously rose, with the highest level occurring from 0–10 min. Then, the increasing trend slowed, as shown in Figure 3; the soluble phosphate increased from 1.1 mg/L to 2.1 mg/L during the first 10 min, and rose slowly to 2.6 mg/L in the following 50 min. This is because part of the soluble phosphate dissolved into overlying water during the disturbance process. On the other hand, the soluble phosphate was easily adsorbed on the suspended particles [15], with a strong adsorption force, which resulted in a slower release.

**Figure 4:**
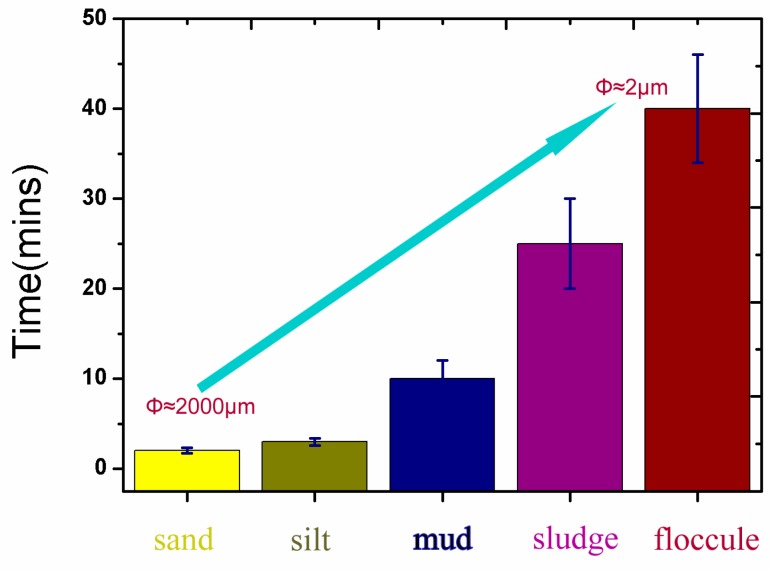
Settling velocity and distribution regularity of the re-suspended matter with different particle sizes (sand, silt ≈ 60–2000 μm; mud, sludge ≈ 5–60 μm; floccule < 2 μm).

**Figure 5:**
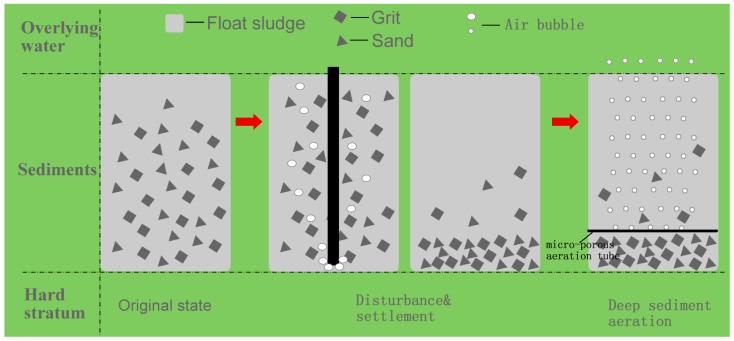
Schematic diagram of the changes of the distribution of particles with different sizes. Large particles, such as grit and sand, settled on the bottom, and the floccule or floated sludge contained many pollutants in the upper layers.

The sediment was completely suspended within 2 to 5 min after sufficient aeration, but the settlement order of the particles showed significant differences because of their density and hydrophobicity. Figure 4 shows that sand and coarse silt subsided within 0–10 min, the mud and sludge layer slowly settled in 10–20 min, and the floccule settled in 30–50 min. The entire process lasted about 3 h, until the thickness of the sediment remained basically unchanged. Finally, the distribution of particles with different sizes in the sediment followed a regular pattern, where large particles, such as grit and sand, settled on the bottom, and the floccule or floated sludge contained many pollutants in the upper layers, as shown in the schematic diagram in Figure 5.

### 3.3. Distribution of Pollutants in Sediment after Settling for 24 h

**Figure 6:**
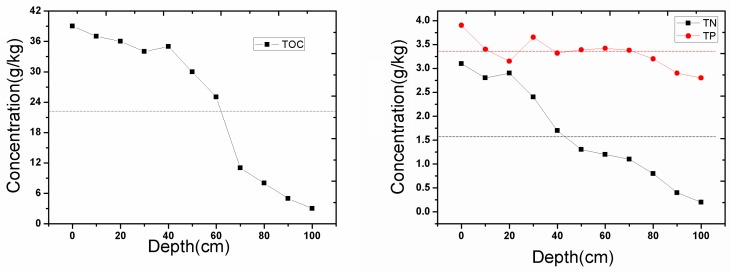
Distributions of TOC, TN, and TP in the sediment after settling for 24 h.

The distribution of organic matter decreased from the top to the bottom, and the mean concentration of the organic matter decreased significantly from 24.1 g/kg to 19.8 g/kg. These results confirmed that aeration disturbance promoted the release of organic matter into the overlying water and formed an orderly distribution of organic matter in the sediment due to the sedimentation process (Figure 6). In addition, the subsidence rate of the organic matter was relatively slow, indicating that the organic matter was absorbed more in the mud and silt than in the sand [28].

The distribution of TN was distributed similar to the organic matter, with the concentration gradually decreasing, as shown in Figure 6. The average TN was 1.6 g/kg, 60% lower than the value before disturbance (3.7 g/kg). Obviously, the sediment disturbance promoted the release of nitrogen from the sediment [11,29]. Another significant change was that the distribution of COD and TN in the sediment became more uniform and decreased with depth, which confirmed that large pieces of impurities, such as sand and grit, were separated from the fluid mud and settled at the bottom. However, the trend of TP was different from that of TOC and TN. The concentration of TP did not significantly change with depth, but had only minor fluctuations, and the average TP (3.3 g/kg) increased slightly compared with the TP before agitation (3.2 g/kg), because the disturbing process of the aeration resulted in an aerobic condition, which inhibited the release of phosphorus from the sediment to the water. Suspended solids and floccules in the water increased significantly in the re-suspension process, and these suspended solids and floccules strengthened the adsorption of phosphorus in the water, and finally stabilized in the sediment [14,30].

### 3.4. Effect of Deep Aeration after Re-Suspending the Sediment

**Figure 7:**
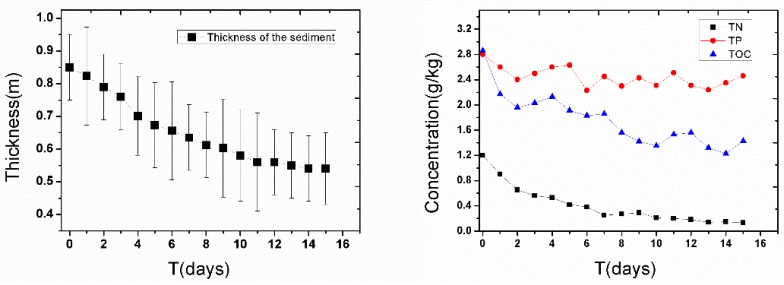
Changes of the sediment thickness and the variation of TN, TP, and TOC in the sediment during 15 days of intermittent sediment aeration.

Intermittent aeration was chosen to save energy and to increase the degradation efficiency. In this process, aeration was conducted for one hour every two hours. After 15 days of aeration, the change of the thickness, and the COD, NH_4_^+^-N, TN, and TP of the sediment are shown in Figure 7. The results showed that the thickness of the sediment reduced from 0.85 m to 0.65 m in the first seven days and slowly reduced to 0.55 m in the following 8 days. This indicated that the sediment aeration promoted the release and degradation of pollution in the sediment and quickly reduced the thickness of the sediment.

Figure 7 shows that the TN and TOC were significantly reduced after 15 days of sediment aeration. The TN declined from 1.20 g/kg to 0.18 g/kg, and the TOC declined from 2.83 g/kg to 1.29 g/kg. This trend was consistent with the increase of the TN and COD in the overlying water (Figure 3 and Figure 4). Compared with TN and TOC, the TP showed slight reduction after 15 days of sediment aeration, and only reduced from 2.81 g/kg to 2.45 g/kg.

Excessive oxygen was supplied both in the surface sediment and in the intermittent water. Most of the oxygen escaped directly from the water in molecular form [26]. Therefore, measures should be taken to increase the efficiency of the supplied oxygen. One possible approach is to increase the contact time of oxygen and pollutants. In this study, we agitated the sediment and allowed it to re-settle, leaving the floating mud and floc with a smaller density and high pollution load to stay on the top of the sediment. The pollutant concentration in this section increased through this re-suspension and settlement process, as shown in Figure 5, and more pollutants were oxidized with oxygen, as shown in Equation (1):

(1) $$C\left(O_{2}\right)+C\left(P\right)↑{(}_{2,settlement}^{1,\;\mathrm{resuspention}})\overset{T↑}{\underset{\left(\mathrm{viscosity}↑\right)}{\to }}C\left(OP\right)↑$$

where C (O_2_), C (P), and C (OP) respectively refer to the concentration of oxygen, pollutants, and the oxidation products.

**Figure 8:**
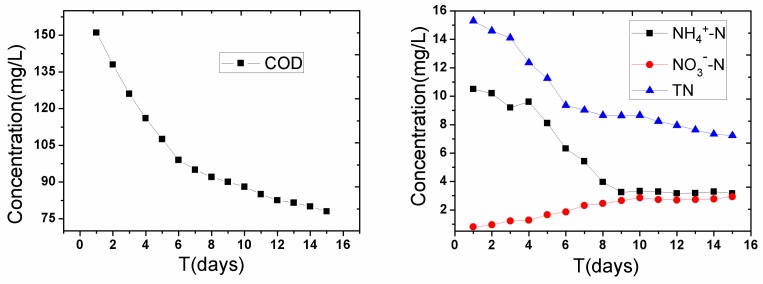
Variation of COD, NH_4_^+^-N, NO_3_^−^-N, and TN in the overlying water during 15 days of intermittent sediment aeration.

Figure 8 shows the changes of COD, NH_4_^+^-N, NO_3_^−^-N, and TN in the overlying water. Compared with the initial state, the COD rapidly decreased from 151 mg/L to 95 mg/L during the first 5 days, and slowly decreased during the next 10 days, because the organic matter, which could be degraded by aerobic microorganisms, degraded during the first stage, and a small proportion of the remaining macro-molecular organics, such as protein, cellulose, and parts of the hydrophobic organic contaminants, degraded during the next stage [13]. Here, the COD was still slightly higher than it was during the initial state because the release speed of the organic matter from the sediment to the water was faster than the degradation speed in the water, and the COD continuously decreased with aeration or sediment adsorption. On the other hand, NH_4_^+^-N showed the largest decrease under the sediment aeration condition (10.5 mg/L to 3.1 mg/L), whereas NO_3_^−^-N increased from 0.3 mg/L to 2.9 mg/L. This indicated that the aeration could effectively prompt the ammonia nitrogen conversion to nitrate nitrogen. The TN also reduced almost 50% compared with its levels at the initial state (15.2 mg/L to 7.6 mg/L).

**Figure 9:**
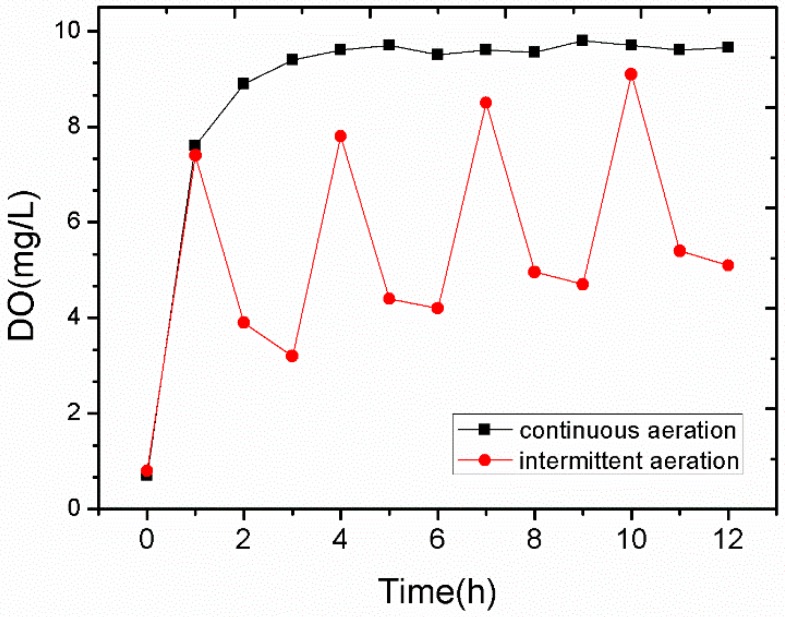
Change of the Dissolved oxygen (DO) during the intermittent aeration process recorded for 12 h.

The ammonia nitrogen will be converted to nitrite nitrogen under aerobic conditions and further converted to nitrate nitrogen with sufficient oxygen, while the nitrite nitrogen will degrade to molecular nitrogen in anaerobic or anoxic conditions [25]. Figure 9 shows the change of DO during the intermittent and continuous aeration process. The DO increased to 9 mg/L in the intermittent aeration process. Periodical fluctuations were observed in the intermittent aeration process, especially in the first hour after stopping the aeration, creating a process of alternating aerobic and anoxic conditions. As such, in the anoxic phase, the denitrification reaction occurred, and parts of the nitrite nitrogen degraded to N_2_.

## 4. Conclusions

Re-suspension of heavily polluted sediments by extreme agitation successfully promoted the release of pollutants, such as organic matter, N, and P in the sediment. The pollutants in the sediment after agitation showed a relatively ordered distribution rather than the random distribution of the initial pre-agitation state. Furthermore, deep sediment aeration after the re-suspension process was successful in reducing the total concentration of contaminants compared to the traditional surface sediment aeration, because of the higher utilization efficiency of oxygen obtained by the longer contact times and the higher concentrations of the reactants. Therefore, using this “re-suspension and deep aeration” method could reduce the risk of black polluted water.

Some of advantages are listed below:

1. The exhaustive re-suspension of sediment promotes the fast release of pollutants in the sediment and uniformly distributes the sediment pollutants. The thickness of the sediments is also reduced.
2. No chemical or biological substances are needed; this causes less destruction of the aquatic ecosystem and is cost effective.
3. Deep sediment aeration results in the high degradation efficiency of pollutants and effectively inhibits the re-release of pollutants in common surface sediment aeration.
